# Reliability Calculation Method of Shipborne Vehicles' Sortie Mission for Dynamic Network Structure

**DOI:** 10.1155/2022/8547961

**Published:** 2022-12-23

**Authors:** Han Shi, Nengjian Wang, Guo Wang, Qinhui Liu, Yuncai Tai

**Affiliations:** ^1^Harbin Engineering University, Harbin, Heilongjiang 150000, China; ^2^Marine Design and Research Institute of China, Shanghai 200000, China

## Abstract

To provide decision support to the commander, it is necessary to calculate shipborne vehicles' sortie mission reliability during the formulation of the layout plan. Therefore, this paper presents the sortie mission network model and reliability calculation method for shipborne vehicles. Firstly, the shipborne vehicle layout and sortie task characteristics are used to establish the sortie mission network model. The shipborne vehicles' sortie mission reliability problem is transformed into a two-terminal network reliability problem. Secondly, the minimal path set method is used to calculate the two-terminal network reliability. An improved tabu search algorithm based on a strategy of breaking up the whole into parts is proposed to search for the minimal path set that matches the length. Finally, the sum of disjoint products is used to process the minimal path set to obtain the shipborne vehicles' sortie mission reliability calculation formula. A numerical analysis of two simplified shipborne vehicles' layouts is given to illustrate the calculation process of the method. This study provides a new evaluation index and an effective quantitative basis for the evaluation system of shipborne vehicles' layout. It also provides theoretical support for the development of decision-making related to the sortie mission of shipborne vehicles.

## 1. Introduction

In amphibious landing operations, shipborne vehicles need to depart from the landing warship's compartment and deliver forces to the designated target area according to the sortie mission instructions [1]. Therefore, to ensure that the shipborne vehicles can depart the landing warship's compartment quickly and safely during landing operations [2], it is necessary to calculate the sortie mission reliability and provide decision support to the commander when formulating the layout plan.

Shipborne vehicles' sortie mission reliability refers to the ability of all vehicles in the layout to leave the landing warship's compartment according to the sortie mission instructions. Before loading shipborne vehicles, the commander needs to formulate a layout plan according to the operational requirements. Specifically, each compartment of the landing warship is the layout space, and the shipborne vehicle is the layout entity. The stowage position in the layout space is the layout plane [3]. An excellent layout plan needs to ensure a sufficient number of assigned loads and that the shipborne vehicle can perform the sortie mission safely, reliably, and efficiently.

The current study on shipborne vehicles' layout is aimed at a higher loading number and obtaining greater deck utilization by compressing the remaining space in the landing warship's compartment [4–7]. At the same time, according to the typical mission style and operational flow of shipborne vehicles, relevant suggestions are given to improve the sortie efficiency [8]. However, during the actual sortie of shipborne vehicles, it may malfunction due to external damage or its performance degradation and even cause the whole layout to be blocked and unable to perform the sortie mission. Therefore, the layout plan is developed to ensure that shipborne vehicles, in the case of partial failure, still have high mission reliability.

At present, there has been much research in the field of related reliability [9–12], in which single-vehicle mission reliability modeling usually uses fault trees analysis, the Markovian model, and the Monte Carlo method [13, 14], while multivehicle mission reliability modeling uses networked models [15, 16]. In the previous research, the generated mission sequence or mission network is determined to be constant when calculating mission reliability. But in the process of shipborne vehicles performing sortie missions, there is a complex spatial relationship between vehicles and vehicles and vehicles and environment with mutual constraints. The sortie of one vehicle will change the sortable state of other vehicles. This change results in an ever-changing sequence of sorties or a network of sortie missions generated by a deployment scheme. If the traditional static modeling method is used, when the previously mentioned changes occur, the shipborne vehicles' sortie mission reliability model needs to be remodeled, which will waste a lot of computation time and resources. Therefore, calculating the shipborne vehicles' sortie mission reliability requires network modeling of vehicle placement, spatial relationships, and sortie sequence. In this way, the dynamic changes of shipborne vehicles' sortie can be simulated by changing the network state. For the dynamic modeling methods, Seung Ki Shin[17] reviewed such methods and related developments including dynamic fault tree, dynamic modeling based on a Petri net, dynamic reliability block diagram, and Monte Carlo simulation based on a time-to-failure tree.

Since the process of the shipborne vehicles executing sortie missions in a layout starts from the first vehicle ready for sortie until the last vehicle leaves the landing warship's compartment, it can be translated into calculating the two-terminal network reliability of the sortie mission network. In the field of two-terminal network reliability research, Vaibhav Gaur [18] reviewed the various algorithms for computing network reliability, including the characteristics, application scenarios, advantages, and limitations of the algorithms in detail. Stefano Sebastio [19] proposed an algorithm for the fast computation of network reliability bounds to find important minimal paths/cuts to quickly reduce the gap between the reliability upper and lower bounds and approximate the exact value. Guanghan Bai [20] developed an ordering heuristic algorithm to improve the efficiency of reliability evaluation. It found that the importance of each minimal path is different, and the ranking of minimal paths according to their importance can improve the computational efficiency of two-terminal network reliability. The previous theories can provide some theoretical basis for the research of shipborne vehicles' sortie mission reliability. Unlike static networks, the changes in spatial constraint relationships will lead to the arc in the network turning on and off, so the existing reliability calculation method for the two-terminal network cannot be utilized directly.

Therefore, based on the characteristics of shipborne vehicles' sortie missions, a dynamic reliability block diagram is firstly adopted to model the sortie mission process. Secondly, an improved search algorithm is proposed to search importantly specified minimal paths, and these paths are sorted in the search process. Finally, these minimal paths are processed to obtain the shipborne vehicles' sortie mission reliability calculation formula. This study will serve as the basis for the shipborne vehicles' sortie mission reliability research, providing a quantitative reference for the layout of shipborne vehicles and theoretical support for subsequent research.

This paper is organized as follows: in Section 2, the characteristics of shipborne vehicles' layout and sortie are analyzed, a reliability model of shipborne vehicles' mission is constructed, and the sortie mission network generation is given. In Section 3, a minimal paths method based on an improved tabu search algorithm is proposed to calculate the two-terminal reliability of the sortie mission network. In Section 4, the calculation process of the method is illustrated by a case study, and the variation law of the mission reliability with different failure rates is summarized. In Section 5, some conclusions and planned future work are given.

## 2. Modeling of Shipborne Vehicles' Sortie Mission Network

In this section, the characteristics of the shipborne vehicles' layouts and the characteristics of the shipborne vehicles' sortie are described first. After that, the shipborne vehicles' sortie mission network (hereinafter referred to as the mission network) model is constructed. In the end, the generation process of the mission network is given.

### 2.1. The Characteristics of Shipborne Vehicles' Layout and Sortie

**Figure 1:**
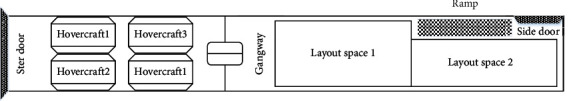
The shipborne vehicles' layout compartment of a landing warship.

Shipborne vehicles are usually arranged in each compartment of the landing warship. To facilitate the loading of shipborne vehicles and the execution of sortie missions, a landing warship is usually equipped with multiple compartments and a complex channel system consisting of side doors, stern doors, gangways, large lifts, and fixed/movable ramps. Taking a landing warship as an example, the shipborne vehicles' layout compartment is shown in Figure 1.

When formulating the layout, relevant safety distances, turning radii, reversing distances, and transfer requirements are usually given priority according to the parameters of shipborne vehicles and layout space, and then the deck utilization rate is the goal to place as many vehicles as possible in the limited space [21, 22]. By the time the shipborne vehicles receive the sortie order, each vehicle executes the sortie mission in turn according to the mission command and the given space constraints (the space constraints are usually the space requirements for the shipborne vehicles to sortie forward, backward, and sideways). Vehicles are transferred from the layout space to the hovercraft via the gangway and then transferred from the hovercraft to the target location via the stern door [23].

**Figure 2:**
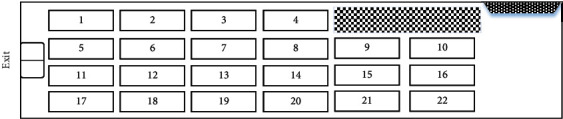
Vehicles' layout *A*.

**Figure 3:**
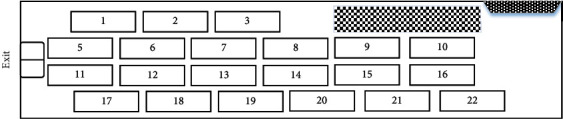
Vehicles' layout *B*.

The sortie characteristics of the shipborne vehicles are analyzed with the layout as shown in Figures 2 and 3. It is assumed that there are 22 vehicles in layout *A* as shown in Figure 2. Due to the lack of sufficient space, the two rows of vehicles near the inner wall of the compartment (No. 1∼No. 4 and No. 17∼No. 22) are temporarily unable to perform the sortie mission, and only the middle two rows of vehicles (No. 5∼No. 10 and No. 11) can be left in order of sortie. Only after the middle two rows of vehicles are both out can the remaining two rows of vehicles start moving from the one near the side door to the one near the gangway.

From the previous analysis, we can see that the layout with the objective of deck utilization extremely restricts the order of vehicles sortie. If the deck utilization and the number of vehicles are reduced appropriately, the transfer space required for vehicles to perform sortie missions can be increased. We are assuming a reduction of one vehicle (No. 4) from layout *A* and arranging the remaining vehicles into layout *B* as shown in Figure 3. As can be seen in Figure 3, after the two vehicles near the exit (No. 5 and No. 11) are out, there is enough transfer space between the two rows of vehicles near the inner wall of the compartment (No. 1∼No. 3 and No. 17∼No. 22) and the exit to get out directly.

From the comparison of layout *A* and layout *B*, it can be seen that when the total number of vehicles is reduced, the spatial limitation between the environment and individual vehicles is weakened, increasing the amount of sortie order available. Therefore, when constructing the mission network model, the spatial relationship between the environment and individual vehicles should be fully considered.

### 2.2. Mission Network Model

To construct the mission network model, the main idea is to express the sortie mission process of shipborne vehicles in the form of an active network. The problem of sortie mission reliability is thus transformed into a two-terminal network reliability problem.

Two-terminal network reliability refers to the probability of being able to connect from the source to the terminal of the network. It is an important part of current network reliability research based on probability theory and supported by reliability graph theory. In reliability graph theory, a network is usually represented by a graph model consisting of a set of nodes and a set of arcs. In the graph model, each arc represents the relationship between the two nodes connected. Also, each arc is assigned a failure probability called the reliability of this arc.

Therefore, the following assumptions are made for the mission network model in conjunction with the previous analysis of shipborne vehicles' layout and sortie mission characteristics:

  Assumption 1: each shipborne vehicle in the layout is independent of the others, and any one of the vehicles' failures will not cause the others to fail
  Assumption 2: nodes in the mission network do not fail
  Assumption 3: arcs in the mission network have only two states, normal and failed
  Assumption 4: the network source node characterizes the shipborne vehicles to start the sortie mission, and the network terminal node characterizes to complete the sortie mission

Combining the previous assumptions, the mission network basic model is constructed as follows:

(1) $$\begin{array}{c} \left\{\begin{array}{c} G=\left(N,A\right)\text{,}\\ N=\left[I,\left(EN,EX\right),O\right]\text{,}\\ A=\left(CS,ES,NW\right)\text{.} \end{array}\right. \end{array}$$

In this network model, *N* is the set of network nodes containing the input vehicles' (already out) information EN, the output vehicles' (soon to be out) information EX, the network source node *I*, and the network terminal node *O*; *A* is the set of network arcs containing the connection status CS, the corresponding vehicle number ES, and the probability of normal operation for this vehicle NW.

Based on the previous model, the spatial constraints of the shipborne vehicles' layout need to be given to reflect the spatial relationships among the individual vehicles and between the environment and the vehicles. These relationships will be mapped to the initial connection states of the arcs in the network and the dynamic changes of the state, which are different in the mission network models corresponding to different shipborne vehicles' layouts.

The spatial constraints used to calculate the state of the arc connection are as follows:

(1) Forward sortie conditions for shipborne vehicles:
  (2) $$\begin{array}{c} \left\{\begin{array}{c} \left|D_{r}\right|>\left|V_{r}^{i}\right|,\left|D_{l}\right|<\left|V_{l}^{i}\right|\text{,}\\ D_{f}^{i}=H\left(\left|E_{f}\right|,\left|V_{f}^{i}\right|,\left|V_{r}^{i}\right|,\left|V_{l}^{i}\right|\right)\text{,}\\ V^{j}=H\left(\left|V_{f}^{j}\right|,\left|V_{l}^{j}\right|,\left|V_{r}^{j}\right|,\left|V_{l}^{j}\right|\right)\text{,}\\ D_{f}^{i}+V^{j}\geq \left|D_{f}^{i}+V^{j}\right|\text{.} \end{array}\right. \end{array}$$
(2) Lateral sortie conditions for shipborne vehicles (forward unobstructed):
  (3) $$\begin{array}{c} \left\{\begin{array}{c} S_{f}^{i}=H\left(\left|E_{f}\right|,\left|V_{f}^{i}\right|,\left|V_{r}^{i}\right|,\left|V_{l}^{i}\right|\right)\text{,}\\ T^{i}=H\left(\left|E_{f}\right|,\left|V_{f}^{i}\right|,\left|V_{z}^{i}\right|,\left|D_{z}\right|\right)\text{,}\\ {\left|S_{f}^{i}\right|}_{l}>\left|V_{l}^{i}\right|\text{,}\\ S_{f}^{i}+V^{j}\geq \left|S_{f}^{i}+V^{j}\right|\text{,}\\ T^{i}+V^{j}\geq \left|T^{i}+V^{j}\right|\text{.} \end{array}\right. \end{array}$$
(3) Lateral sortie conditions for shipborne vehicles (forward facing with cover):
  (4) $$\begin{array}{c} \left\{\begin{array}{c} V_{fm}^{i}=H\left(\left|V_{f}^{i}\right|+{\left|V^{i}\right|}_{l},\left|V_{f}^{i}\right|,\left|V_{r}^{i}\right|,\left|V_{l}^{i}\right|\right)\text{,}\\ V_{sm}^{i}=H\left(\left|V_{f}^{i}\right|,\left|V_{b}^{i}\right|,\left|V_{r}^{i}\right|+n{\left|V^{i}\right|}_{w},\left|V_{l}^{i}\right|+n{\left|V^{i}\right|}_{w}\right)\text{,}\\ \left|E_{r}\right|>{\left|V_{sm}^{i}\right|}_{w}^{+},\left|E_{l}\right|<{\left|V_{sm}^{i}\right|}_{w}^{-}\text{,}\\ V_{fm}^{i}+V^{j}\geq \left|V_{fm}^{i}+V^{j}\right|\text{,}\\ V_{sm}^{i}+V^{j}\geq \left|V_{sm}^{i}+V^{j}\right|\text{,}\\ V_{sm}^{i}\Rightarrow \left(2\right)\vee \left(3\right)\text{.} \end{array}\right. \end{array}$$
(4) Backward sortie conditions for shipborne vehicles:
  (5) $$\begin{array}{c} \left\{\begin{array}{c} V_{bm}^{i}=H\left(\left|V_{b}^{i}\right|,\left|V_{b}^{i}\right|+{\left|V^{i}\right|}_{l},\left|V_{r}^{i}\right|,\left|V_{l}^{i}\right|\right)\text{,}\\ V_{bm}^{i}+V^{j}\geq \left|V_{bm}^{i}+V^{j}\right|\text{,}\\ V_{bm}^{i}\Rightarrow \left(4\right)\text{.} \end{array}\right.<listaend><listaend> \end{array}$$

The symbols in the constraints are described as follows:

1. *i* is the vehicle serial number, and *j* is the serial number of all vehicles other than the *i*th vehicle
2. *H*(*f*, *b*, *r*, *l*) denotes the rectangle consisting of *x*_1_=*f*, *x*_2_=*b*, *y*_1_=*r*, *y*_2_=*l*
3. *D*_*r*_ denotes the boundary to the right of the exit when the vehicle is facing the exit, and |*D*_*r*_| denotes the *Y*-axis coordinate of the boundary to the right of the exit
4. *D*_*l*_ denotes the boundary to the left of the exit when the vehicle is facing the exit, and |*D*_*l*_| denotes the *Y*-axis coordinate of the boundary to the left of the exit
5. *V*_*r*_^*i*^ denotes the boundary to the right of the *i*th piece of the vehicle as it faces the exit, and |*V*_*r*_^*i*^| denotes the *Y*-axis coordinate of the boundary to the right of the vehicle
6. *V*_*l*_^*i*^ denotes the boundary to the left of the *i*th piece of the vehicle as it faces the exit, and |*V*_*l*_^*i*^| denotes the *Y*-axis coordinate of the boundary to the left of the vehicle
7. *E*_*f*_ denotes the compartment boundary at which the exit is located, and |*E*_*f*_| denotes the *X*-axis coordinates of the compartment boundary
8. *V*_*f*_^*i*^ denotes the boundary to the front side of the ith piece of the vehicle as it faces the exit, and |*V*_*f*_^*i*^| denotes the *X*-axis coordinate of the boundary to the front of the vehicle
9. *V*_*b*_^*i*^ denotes the boundary to the rear side of the *i*th piece of the vehicle as it faces the exit, and |*V*_*b*_^*i*^| denotes the *X*-axis coordinate of the boundary to the rear of the vehicle
10. |*S*_*f*_^*i*^|_*l*_ denotes the length of the in the *X*-axis direction
11. *V*_*z*_^*i*^ denotes the centroid of the *i*th vehicle, and |*V*_*z*_^*i*^| denotes the *Y*-axis coordinates of the vehicle centroid
12. *D*_*z*_ denotes the centroid of the exit, and |*D*_*z*_| denotes the *Y*-axis coordinates of the exit
13. |*V*^*i*^|_*l*_ denotes the length of the *V*^*i*^ in the *X*-axis direction, and |*V*^*i*^|_*w*_ denotes the length of the *V*^*i*^ in the *Y*-axis direction
14.  *E*_*r*_ denotes the cabin boundary on the right side of the exit, and |*E*_*r*_| denotes the *Y*-axis coordinates of the boundary on the right side of the cabin
15. *E*_*l*_ denotes the cabin boundary on the right side of the exit, and |*E*_*l*_| denotes the *Y*-axis coordinates of the boundary on the right side of the cabin
16. ⇒ indicates that the symbol on the left side satisfies the constraint on the right side

Also, for the convenience of subsequent calculations, the following definition is made for the mission network model.

**Definition 1 .**

If an arc is connected between *N*_*i*_ and *N*_*j*_ and the direction of the arc points from *N*_*i*_ to *N*_*j*_, then *N*_*j*_ is a subnode of *N*_*i*_, the set of *N*_*j*_ is a subset of *N*_*i*_ (*i*=1,2, ⋯, *m*, *j*=1,2, ⋯, *m*, and *m* is the total number of vehicles, *i* ≠ *j*). We denote

(6) $$\begin{array}{c} \left\{\begin{array}{c} N_{i}⟶N_{j}\text{,}\\ Nc\left(N_{i}\right)=\left\{N_{j}\right\}\text{.} \end{array}\right. \end{array}$$

**Definition 2 .**

If there is a sequence of arcs of length *n* that can reach *N*_*j*_ from a specified node *N*_*i*_ and if this arc sequence is a minimal path from *N*_*i*_ to *N*_*j*_ and the vehicle information of all arcs in the arc sequence does not repeat, then this arc sequence is a fixed-length minimal path from *N*_*i*_ to *N*_*j*_.

### 2.3. Mission Network Generation Process

Based on the mission network model, the mission network generation process is determined as follows:

1. Initialization: the shipborne vehicles' sortie mission network consists of the sortie order and the shipborne vehicles in the layout. According to the reliability block diagram theory, the sortie order is mapped to the nodes of the network, and the shipborne vehicles' information is mapped to the arcs. A node is generated between every two pieces of the vehicle. This node represents the order in which these two pieces of the vehicle are out to each other. The total number of network nodes is determined by increasing the input and output node.
2. Connection and assignment: if there is an intersection of the vehicle sortie order represented by the two nodes, the two nodes are connected by an arc. According to the given space constraints of the shipborne vehicles' layout, we calculate the vehicles' operation condition represented by the arc and determine the connection type of the arc. If the vehicle represented by the current arc does not meet the departure condition, it is a dashed connection, and vice versa, it is a solid connection. The result of the calculation, together with the vehicle number and normal operation probability, is packaged and assigned to the arc.
3. Connection of input and output node: the initial deployable vehicle of the shipborne vehicles' layout is calculated by connecting the input nodes to the nodes containing these vehicles in one direction and calculating whether the output vehicle of these nodes satisfies the subsequent deployable conditions. If it is satisfied, it is a solid connection, and vice versa, it is a dashed connection. The output node is connected to all nodes except for the input node with dashed lines.
4. Simulation of the layout change: the simulation of the layouts' dynamic change is changed by changing the connection state of the arc. If the arc is a solid connection, the arc becomes a dashed connection after the vehicle is out; if the arc is a dashed connection, the arc becomes a solid connection after the outgoing space constraint is satisfied. When the first vehicle went out, all the arcs connected to the input node become dashed connections and when the last vehicle is ready to move, the arcs connected to the output node become solid connections.
5. Outputs: we output the generated network.

## 3. Reliability Calculation Method of Shipborne Vehicles' Sortie Mission

By constructing the mission network model, the problem of shipborne vehicles' sortie mission reliability is transformed into the problem of calculating the tow-terminal reliability of the mission network. Therefore, this paper uses the minimal path set method to calculate the two-terminal reliability of the mission network based on the mission network model constructed by the dynamic reliability block diagram and the shipborne vehicles' sortie characteristics.

The minimal path set method is a classical algorithm for solving network reliability, mainly by searching for the minimal path between two specified nodes in the network to solve the probability of connectivity between two nodes. If the arc in the minimal path is not repaired after failure, the product of the probability that all arcs work properly is the probability that the minimal path is connected. The probability that all the minimal paths in the minimal path sets are connected is the reliability between the specified two nodes in the network. If we note that *A*_1_, *A*_2_, ⋯, *A*_*m*_ are all the minimal paths from the input node to the output node of the network, the two-terminal network reliability is calculated as follows:

(7) $$\begin{array}{c} R=P\left\{\overset{m}{\underset{i=1}{\cup }}A_{i}\right\}\text{.} \end{array}$$

Since there is a certain intersection of the minimal paths in the minimal path set, the sum of disjoint products is now generally used to treat Equation (7). According to the Boolean algebraic rule, for two intersecting minimal paths *A*_*i*_ and *A*_*j*_, *i* ∈ [1, *m*], *j* ∈ [1, *m*], *i* ≠ *j*, the two minimal paths after disjoint are as follows:

(8) $$\begin{array}{c} A\cup B=A+B=A+\bar{A}B\text{.} \end{array}$$

$\bar{A}$ denotes an event that occurs in *A* but not in *B*.

Therefore, in this section, a suitable minimal path search algorithm is first selected based on the shipborne vehicles' sortie characteristics and the mission network model. Then, it is improved by using breaking up the whole into parts' strategy. Finally, the minimal path set that meets the length of the search is disjoint, and the equation for calculating the mission reliability of shipborne vehicles' sorties is obtained according to the characteristics of the fixed-length minimal path.

### 3.1. Search Algorithm Selection

The common algorithms for searching the minimal path are mainly enumerative and heuristic search algorithms. The enumeration method searches for all the minimal paths between the input node and the output node by traversing all the nodes in the network, but it can be computationally intensive and slow, and the minimal path set searched needs to be further sorted according to independence with a large amount of computational redundancy. The heuristic search algorithm prioritizes the search for the minimal path that contributes to the reliability calculation. This algorithm will remove the corresponding arcs in the network after one iteration to ensure the highest independence of the solutions searched for each time. It solves the drawback of computational redundancy of the enumeration method, but the operation of deleting arcs leads to a limited number of minimal paths for its search. To search the minimal paths more efficiently, we omit the minimal paths that do not match the length, reduce the amount of computation and occupied resources, and avoid the operation of deleting arcs when the heuristic search algorithm deletes the searched minimal paths, the tabu search algorithm is proposed to search the fixed-length minimal path set of the mission network.

The traditional tabu search algorithm starts from an initial solution and chooses a series of specific search directions to move in and maximize the change in the value of the objective function. The algorithm simulates the human thought process in the search to avoid getting trapped in a local optimal solution, and the search process that has been performed is recorded to guide the next move. Compared with the traditional minimal path search algorithm, the advantages of the tabu search algorithm lie in the following points:

1. As long as the given initial solution and fitness value function is suitable, the fixed-length minimal path can be searched quickly, and the searched results do not need to be sorted
2. With the addition of the tabu list, the fixed-length minimal paths with more repeated arcs are not obtained in the first few rounds of the search
3. The tabu length and the aspiration criterion are added so that the local optimal solution will not be trapped in the later rounds of the search

However, the traditional tabu search algorithm still searches based on the initial solution, and it is difficult to quickly find the ideal solution that contributes most to the reliability calculation by ordinary moves (interchange, insertion, inverse order, etc.). To make the tabu search algorithm obtain the ideal solution quickly, this article improves the tabu search algorithm by dividing the solution space into multiple subspaces and directing the tabu search to move in a specific direction in each subspace using the breaking up the whole into parts' strategy.

### 3.2. A Tabu Search Algorithm Based on Breaking up the Whole into Parts' Strategy

The strategy of breaking up the whole into parts refers to the practice of reducing what is difficult to achieve in one negotiation into subelements with subgoals and achieving the subgoals to meet the overall goal. The usual steps for breaking up the whole into parts' strategy are as follows:

(1) Determination of the decomposition content: according to the nature of the negotiation content, the minimal unit after decomposition is developed. The negotiations in this paper are fixed-length minimal paths, and the decomposed minimal units are arcs.
(2) Decomposition of negotiation content: the negotiation content is decomposed according to the structure. Each item can be independent as negotiation content and has corresponding subgoals, and the decomposition needs to facilitate the realization of the overall goal and refine the overall goal. In this article, we need to decompose the fixed-length minimal path into independent arcs, and each arc has its neighborhood, subdomain, and subadaptation value objectives.
(3) Organization of negotiations:
  ① Organization of negotiations for each unit: determine the corresponding negotiation direction according to the subgoals of each unit
  ② Controlling the objectives of each unit: each unit has different levels of difficulty in the negotiation, and multiple unit subobjectives are needed to complement each other to ensure the achievement of the overall objectives
  ③ Cleaning of strategy results: to achieve the overall goal, the total benefits of each subgoal need to be combined to determine whether the initial goal has been achieved

In the search process of this problem, it is necessary to first decide whether the current search subspace is an arc neighborhood or a subdomain, then move or expand according to the guiding direction of the subfitness value function, and finally judge whether the new solution satisfies the total fitness value objective.

Before using the breaking up the whole into parts' strategy to improve the tabu search algorithm, the following definitions are made:

Let there be arcs *f*_*i*_ and *f*_*j*_ in the initial solution and arcs *f*_*i*_′ and *f*_*j*_′ in the new solution.

**Definition 3 .**

We define the operator “+=” as connected. If the input node of *f*_*j*_ and the output node of *f*_*i*_ are the same, then *f*_*j*_ is connected to *f*_*i*_. The mathematical representation is as follows:

(9) $$\begin{array}{c} f_{i}+=f_{j}\text{.} \end{array}$$

**Definition 4 .**

We define the operator “+≠” as disconnected. If the input node of *f*_*j*_ and the output node of *f*_*i*_ are different, then *f*_*j*_ is disconnected from *f*_*i*_. The mathematical representation is as follows:

(10) $$\begin{array}{c} f_{i}+\neq f_{j}\text{.} \end{array}$$

**Definition 5 .**

If *f*_*j*_ is connected to *f*_*i*_, then the neighborhood of *f*_*i*_ is a subdomain of *f*_*i*_. The mathematical representation is as follows:

(11) $$\begin{array}{c} \left\{\begin{array}{c} Nh\left(f_{j}\right)\in Ch\left(f_{i}\right)\text{,}\\ Ch\left(f_{i}\right)=Nh\left(\left\{f_{j}\right\}\right)\text{.} \end{array}\right. \end{array}$$

**Definition 6 .**

Known *f*_*i*_′ ∈ *Nh*(*f*_*i*_), *f*_*i*_′+=*f*_*j*_′, if *f*_*j*_′ ∈ *Nh*(*f*_*j*_), then the action from *f*_*j*_ to *f*_*j*_′ is recorded as a move; if *f*_*j*_′ ∉ *Nh*(*f*_*j*_), then the action from *f*_*j*_ to *f*_*j*_′ is recorded as an expansion.

The basic elements of the tabu search algorithm based on the breaking up the whole into parts' strategy are determined according to the previous steps and definitions as follows:

(1) Initial solution: it is a randomized, fixed-length minimal path in the mission network.
(2) Objective function: the objective function of the algorithm is divided into a total objective function and a subobjective function. The subobjective function guides the movement or expansion of the solution, and the general objective function is used to determine whether a solution satisfies the initial objective.
The subobjective function *y*_*s*_ is mathematically represented as follows:
  (12) $$\begin{array}{c} y_{s}=card\left\{\left.f\right|f\notin Q\right\}\leq 1\text{.} \end{array}$$
The total objective function *y*_*m*_ is mathematically represented as follows:
  (13) $$\begin{array}{c} y_{m}=card\left\{\left.f\right|f\in F\vee f\notin Q\right\}\leq n=Length\left({Initial}_{solution}\right)\text{.} \end{array}$$
(3) Neighborhood and movement: the neighborhood of an arc *f*_*i*_ is the set of arcs that are the same as the input node of *f*_*i*_, and the modification of the solution on its neighborhood is movement.
(4) Subdomain and augmentation: the subdomain of an arc *f*_*i*_ is the set of arcs with the output node of *f*_*i*_ as the input node, and the modification of the solution on its subdomain is augmentation.
(5) Tabu object, tabu list, and tabu length: the tabu object is the arc that forms a fixed-length minimal path, the tabu list is the neighborhood and subdomain of the arc, and the tabu length is the length of the tabu list.
(6) Termination condition: the number of termination iterations of the algorithm is calculated from the given reliability calculation error.

From the previous basic elements, it is clear that before searching for a fixed-length minimal path using the tabu search algorithm based on the breaking up the whole into parts' strategy, the initial solution needs to be searched for. Firstly, using depth-first search, a child node *N*_1_ of the input node *I* of the mission network is randomly selected, the connected arc *f*_1_ is recorded, and the network state is updated. Then, the child node *N*_2_ of the node *N*_1_ is randomly selected, the connected arc *f*_2_ is recorded, and the network status is updated. The previous action is next repeated until node *N*_*n*−1_ and arc *f*_*n*−1_ are found, at which point the arc *f*_*n*_ between node *N*_*n*−1_ and output node *O* is activated. Finally, the arc *f*_*n*_ is recorded, and the whole fixed-length minimal path *F*=(*f*_1_, *f*_2_, ⋯, *f*_*n*_) is output to obtain the initial solution *F* recorded in the total solution set *Q*.

After the initial solution is obtained, the flow of the tabu search algorithm based on breaking up the whole into parts' strategy is as follows:

Step 1: dividing the solution *F* into *n* segments according to the breaking up of the whole into parts' strategy so that *i*=1 and *m*=0. (Here, *n* is the number of shipborne vehicles in the layout, *i* is the serial number of the current arc in the solution, and *m* is the condition to judge the arc movement or expansion.)
Step 2: judgment of the search criteria:
    Condition 1: if *m*=0, then a neighborhood *Nh*(*f*_*i*_) of *f*_*i*_ is generated such that *f*_*i*_ moves along the direction guided by the subobjective function to get *f*_*i*_′
    Condition 2: if *m*=1, then a subdomain *Ch*(*f*_*i*−1_′) of *f*_*i*−1_′ is generated such that *f*_*i*_ moves along the direction guided by the subobjective function to obtain *f*_*i*_′
Step 3: updating the tabu list and record *f*_*i*_′ in *F*′.
Step 4: judgment of the end of the search round conditions:
    Condition 1: if *i*=*n*, then let *F*=*F*′, turn Step7
    Condition 2: if *i* ≠ *n*, then let *i*=*i*+1, *m*=0
Step 5: the neighborhood *Nh*(*f*_*i*_) of *f*_*i*_ is generated such that *f*_*i*_ moves along the direction guided by the subobjective function to get *f*_*i*_′.
Step 6: judgment on whether this movement is reasonable:
    Condition 1: if *f*_*i*−1_′+=*f*_*i*_′, then update the tabu list and record *f*_*i*_′ in *F*′, turn Step 4
    Condition 2: if *f*_*i*−1_′+≠*f*_*i*_′, then let *m*=1, turn Step 2
Step 7: judgment of the termination condition of the algorithm:
    Condition 1: if the termination condition is not satisfied, then we write down the solution *F* into the total solution set *Q*, update the objective function according to the *Q*, reset the mission network, and turn Step 1
    Condition 2: if the termination condition is satisfied, then the search is terminated and the total solution set *Q* is output

At this point, the total solution set *Q* is the required fixed-length minimal path set.

### 3.3. Disjoint Processing of Fixed-Length Minimal Paths

From equations (7) and (8), it can be seen that when the fixed-length minimal path set *Q* of the shipborne vehicles' sortie mission network is known, the calculation of the sortie mission reliability requires the disjoint processing of the searched fixed-length minimal path set. Therefore, if we note *a*_*i*_=(*a*_1_, *a*_2_, ⋯, *a*_*m*_), *i*=1,2, ⋯, *m*, the event in Equation (8) that occurs in *A* but not in *B*, then

(14) $$\begin{array}{c} A+B=A+\bar{A}B\\ =A+\bar{A-B}B\\ =A+\bar{a_{1}}B+a_{1}\bar{a_{2}}B+\cdots +a_{1}a_{2}\cdots \bar{a_{m}}B\\ =A+\left(\bar{a_{1}}+a_{1}\bar{a_{2}}+\cdots +a_{1}a_{2}\cdots \bar{a_{m}}\right)B\text{.} \end{array}$$

Expanding equation (7) according to equation (14), we get

(15) $$\begin{array}{c} R=P\left\{A_{1}+\bar{A_{1}}A_{2}+\bar{A_{1}A_{2}}A_{3}+\cdots \bar{A_{1}A_{2}\cdots A_{i-1}}A_{i}\right\}\\ =P\left\{A_{1}\right\}+P\left\{\bar{A_{1}}A_{2}\right\}+\cdots +P\left\{\bar{A_{1}A_{2}\cdots A_{i-1}}A_{i}\right\}\text{.} \end{array}$$

Therefore, if *F*_1_, *F*_1_, ⋯, *F*_*k*_ is set to be the minimal path of all fixed-lengths in the mission network *G*, then the shipborne vehicles' sortie mission reliability is as follows:

(16) $$\begin{array}{c} R_{I,O}\left(G\right)=P\left(F_{1}+F_{2}+\cdots +F_{k}\right)\\ =P\left(F_{1}\right)+P\left(\bar{F_{1}}F_{2}\right)+\cdots +P\left(\bar{F_{1}}\bar{F_{2}}\cdots \bar{F_{k-1}}F_{k}\right)\text{.} \end{array}$$

According to equation (14), equation (16) can be expanded as follows:

(17) $$\begin{array}{c} R_{I,O}\left(G\right)=P\left(F_{1}\right)+P\left(\bar{F_{1}-F_{2}}F_{2}\right)+P\left(\bar{F_{1}-F_{3}}\bar{F_{2}-F_{1}F_{3}}F_{3}\right)+\cdots +P\left(\bar{F_{1}-F_{k}}\bar{F_{2}-F_{1}F_{k}}\cdots \bar{F_{k-1}-F_{1}F_{2}\cdots F_{k-2}F_{k}}F_{k}\right)\\ =P\left(F_{1}\right)+P\left(\bar{F_{1}-F_{2}}\right)P\left(F_{2}\right)+P\left(\bar{F_{1}-F_{3}}\bar{F_{2}-F_{1}F_{3}}\right)P\left(F_{3}\right)+\cdots +P\left(\bar{F_{1}-F_{k}}\bar{F_{2}-F_{1}F_{k}}\cdots \bar{F_{k-1}-F_{1}F_{2}\cdots F_{k-2}F_{k}}\right)P\left(F_{k}\right)\text{.} \end{array}$$

If *X*_1_, *X*_2_, ⋯, *X*_*n*_ is the probability that each vehicle works properly, then the definition of fixed-length minimal path shows that

(18) $$\begin{array}{c} P\left(F_{1}\right)=P\left(F_{2}\right)=P\left(F_{3}\right)=\cdots =P\left(F_{n}\right)=\overset{n}{\underset{i=1}{\prod }}X_{i}\text{.} \end{array}$$

Therefore, equation (17) can be reduced to

(19) $$\begin{array}{c} R_{I,O}\left(G\right)=P\left(F\right)\left[1+P\left(\bar{F_{1}-F_{2}}\right)+P\left(\bar{F_{1}-F_{3}}\bar{F_{2}-F_{1}F_{3}}\right)+\cdots +P\left(\bar{F_{1}-F_{k}}\bar{F_{2}-F_{1}F_{k}}\cdots \bar{F_{k-1}-F_{1}F_{2}\cdots F_{k-2}F_{k}}\right)\right]. \end{array}$$

If we note that *f*_*i*_=(*f*_1_, *f*_2_, ⋯, *f*_*z*_) is an arc that occurs in *F*_*a*_ but not in *F*_*b*_ (*i*=1,2, ⋯, *z*), then

(20) $$\begin{array}{c} P\left(\bar{F_{a}-F_{b}}\right)=P\left(\bar{f_{1}}\right)+P\left(f_{1}\bar{f_{2}}\right)+\cdots P\left(f_{1}f_{2}\cdots \bar{f_{z}}\right). \end{array}$$

In equation (20), the vehicle information represented by each arc is independent. $P\left(f_{1}f_{2}\cdots \bar{f_{z}}\right)<1\times 10^{1-z}$ because of *P*(*f*_*i*_) < 1, $P\left(\bar{f_{i}}\right)=1-P\left(f_{i}\right)<1$. So, $P\left(\bar{F_{a}-F_{b}}\right)<1$. If $\bar{F_{1}-F_{j}}$, $\bar{F_{2}-F_{1}F_{j}}$, ⋯, $\bar{F_{j-1}-F_{1}F_{2}\cdots F_{j-2}F_{j}}$ are all independent of each other(1 < *j* < *k*), then

(21) $$\begin{array}{c} P\left(\bar{F_{1}-F_{j}}\bar{F_{2}-F_{1}F_{j}}\cdots \bar{F_{j-1}-F_{1}F_{2}\cdots F_{j-2}F_{j}}\right)\\ =P\left(\bar{F_{1}-F_{j}}\right)P\left(\bar{F_{2}-F_{1}F_{j}}\right)\cdots P\left(\bar{F_{j-1}-F_{1}F_{2}\cdots F_{j-2}F_{j}}\right)<1\times 10^{1-j}\text{.} \end{array}$$

From equation (21), it can be seen that as *j* increases, the order of *P* gradually increases, but the order of magnitude gradually decreases, and the impact on the reliability of the calculation results decreases exponentially.

When searching for the fixed-length minimal paths using the tabu search algorithm based on the breaking up the whole into parts' strategy, the minimal paths that are preferentially searched have the greatest variability and are all maximal *n* due to the presence of the total objective function. Thus, $\bar{F_{1}-F_{j}}$, $\bar{F_{2}-F_{1}F_{j}}$, ⋯, $\bar{F_{j-1}-F_{1}F_{2}\cdots F_{j-2}F_{j}}$ are independent of each other when the order is small and has the greatest influence on the reliability calculation; when the order is large, they are not independent of each other but can be approximately regarded as independent of each other because they have minimal influence on the reliability calculation.

If we note $\tilde{F_{c}-F_{d}}=\bar{F_{c}-F_{1}F_{2}\cdots F_{c-1}F_{d}}$, (*c* < *d*), then equation (19) can be reduced to the following:

(22) $$\begin{array}{c} R_{I,O}\left(G\right)=\left(\overset{n}{\underset{i=1}{\prod }}X_{i}\right)\left\{1+\overset{k}{\underset{i=2}{\sum }}\left[\overset{i-1}{\underset{j=1}{\prod }}P\left(\tilde{F_{j}-F_{i}}\right)\right]\right\}\text{.} \end{array}$$

Equation (22) is the shipborne vehicles' sortie mission reliability calculation equation.

## 4. Case Study

To illustrate the process of calculating the mission reliability of shipborne vehicles, two simplified shipborne vehicles' layouts with the characteristics described in chapter II are given and the calculation process is shown. The mission network model, the tabu search algorithm based on the rounding-to-zero strategy, and the reliability equation after the disjointing process were programmed using VS2015 and C++ to calculate the mission reliability of shipborne vehicles under different failure rates for two simplified layout scenarios, respectively, and we briefly analyze the results.

### 4.1. Description of the Typical Layout

We assume that a landing warship is prepared for an amphibious landing mission and needs to carry 9 vehicles. Before the shipborne vehicles are loaded, the commander designs two layouts, and we need to calculate the shipborne vehicles' sortie mission reliability for the commander's decision for each layout. In layout Plan A, nine vehicles are loosely arranged in the layout area. This arrangement reduces the utilization of layout space, but retains sufficient transfer space for shipborne vehicles' sortie. In layout Plan B, nine vehicles are compactly arranged near the exit of the layout area. This arrangement improves the utilization of layout space, but compresses the transfer space when the shipborne vehicles are sortied.

**Figure 4:**
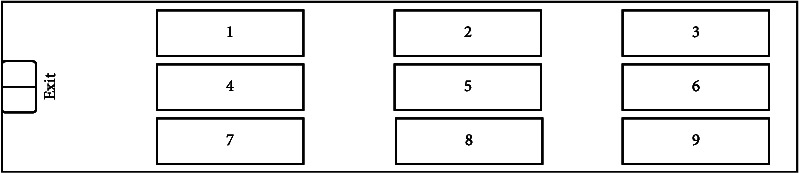
Layout plan *A*.

**Figure 5:**
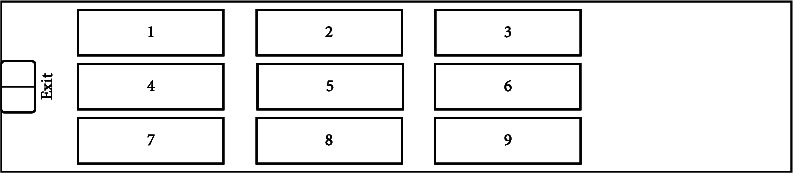
Layout plan *B*.

The specific parameters of the compartment are 50 m long and 14 m wide, the exit is located in the middle of the compartment on the left side, and the width of the exit is 5 m. The specific parameters of the vehicles are 10 m long and 4 m wide, and the number is 9. The coordinates of the vehicles in the two layouts are shown in Table 1:

The two layouts are shown in Figures 4 and 5:

### 4.2. Sortie Mission Reliability Calculation

**Figure 6:**
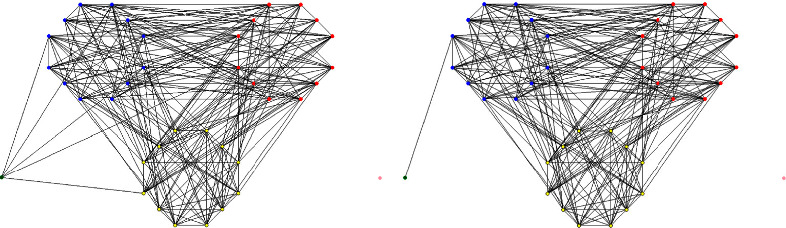
Shipborne vehicles' sortie mission network. (a) Plan *A* mission network. (b) Plan *B* mission network.

Firstly, according to the shipborne vehicles' sortie mission network generation process, the mission networks of Plans *A* and *B* are constructed, as shown in Figure 6. Then, the tabu search algorithm based on breaking up the whole into parts' strategy searches for fixed-length minimal path sets *Q*(*A*) and *Q*(*B*). Finally, the mission reliability of the shipborne vehicles for each layout scenario is calculated according to equation (22) for a low failure rate (1%–10%) and a high failure rate (10%–60%), and the reliability error is required to be controlled within 1 × 10^−6^.

The calculated shipborne vehicles' sortie mission reliability for Plan *A* and Plan *B* is obtained in Tables 2 and 3.

### 4.3. Analysis of Results

**Figure 7:**
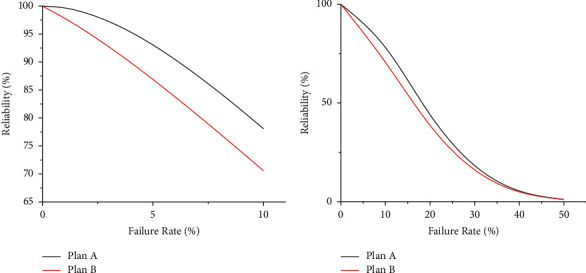
The shipborne vehicles' sortie mission reliability curve. (a) The case of the low failure rate. (b) The case of the high failure rate.

The data from Tables 2 and 3 are plotted as shown in Figure 7.

As can be seen from Figure 7, the loose arrangement has higher reliability than the compact arrangement as the failure rate increases by 10%. At low failure rates, improving the layout form can effectively improve the reliability of the sortie mission, and the benefits gained from improving the layout form gradually increase as the failure rate increases. However, as the failure rate continues to increase, the benefit of mission reliability obtained by improving the layout form does not always increase, but first increases and then decreases, and at a 60% failure rate, the vehicles' sortie reliability in both layouts is close to zero. This indicates that improving the shipborne vehicles' sortie mission reliability only by improving the layout form is more effective in the case of a low failure rate (the actual failure rate threshold is determined by the deployment scheme); in the case of a high failure rate, improving the shipborne vehicles' sortie mission reliability should try to improve the vehicles' security capability and reduce the possibility of vehicles' failure.

## 5. Conclusion

To fully consider the shipborne vehicles' sortie mission reliability during the formulation of the layout, this article proposes a network modeling method according to the shipborne vehicles' layout and sortie mission characteristics, which transforms the shipborne vehicles' sortie mission reliability problem into a two-terminal network reliability problem. In calculating the two-terminal reliability of the mission network, the tabu search algorithm is proposed to be improved by using the breaking up the whole into parts' strategy to obtain the sorted fixed-length minimal path set quickly, and the equation for calculating the reliability of the shipborne vehicles' sortie mission is obtained by processing the fixed-length minimal path set according to the sum of the disjoint products theorem. This article combines the previous network model and calculation methods to calculate the shipborne equipment sortie mission reliability for two layout scenarios, and the calculation results verify the effectiveness of the previous methods. This study can also be extended to the sortie reliability of layout equipment in generally enclosed spaces.

However, there are limitations to the assumption of independent failure rates of shipborne vehicles in the mission network model of this article. The failure rate of other vehicles may be affected when the failed vehicle is not removed evenly, which is beyond the scope of the present research focus. Thus, in future work, these effects will be considered in a more complete reliability assessment model. Also, we will conduct a dispatch study on shipborne vehicle breakdowns during sorties.

## Figures and Tables

**Table 1:** Coordinate vehicle center points for each layout.

Layout	No. 1	No. 2	No. 3	No. 4	No. 5	No. 6	No. 7	No. 8	No. 9
Plan *A*	(15, 11.5)	(29, 11.5)	(43, 11.5)	(15, 7)	(29, 7)	(43, 7)	(15, 2.5)	(29, 2.5)	(43, 2.5)
Plan *B*	(9, 11.5)	(21, 11.5)	(33, 11.5)	(9, 7)	(21, 7)	(33, 7)	(9, 2.5)	(21, 2.5)	(33, 2.5)

*Note.* The lower left corner of the compartment is the coordinate origin, the horizontal is the *x*-axis, and the vertical is the *y*-axis.

**Table 2:** Shipborne vehicles' sortie mission reliability with the low failure rate.

Layout	1%	2%	3%	4%	5%	6%	7%	8%	9%	10%
Plan *A*	0.996721	0.987184	0.972434	0.953338	0.930432	0.904496	0.876078	0.845651	0.813774	0.780668
Plan *B*	0.978411	0.954046	0.927392	0.898940	0.868923	0.837715	0.805561	0.772684	0.739358	0.705714

**Table 3:** Shipborne vehicles' sortie mission reliability with the high failure rate.

Layout	10%	20%	30%	40%	50%
Plan *A*	0.780668	0.440260	0.182918	0.054471	0.011383
Plan *B*	0.705714	0.386126	0.160914	0.049417	0.010749

## Data Availability

The experimental data used to support the findings of this study are available from the corresponding author upon request.
